# Exploring the use of leucine zippers for the generation of a new class of inclusion bodies for pharma and biotechnological applications

**DOI:** 10.1186/s12934-020-01425-x

**Published:** 2020-09-04

**Authors:** Ramon Roca-Pinilla, Sara Fortuna, Antonino Natalello, Alejandro Sánchez-Chardi, Diletta Ami, Anna Arís, Elena Garcia-Fruitós

**Affiliations:** 1 Department of Ruminant Production, Institute of Agriculture and Food Research and Technology (IRTA), 08140 Caldes de Montbui, Spain; 2 Department of Chemical and Pharmaceutical Sciences, University of Trieste, Via L. Giorgieri 1, 34127 Trieste, Italy; 3 Department of Biotechnology and Biosciences, University of Milano-Bicocca, 20126 Milan, Italy; 4 Department of Evolutionary Biology, Ecology and Environmental Sciences, Faculty of Biology, University of Barcelona (UB), 08028 Barcelona, Spain; 5 Microscopy Service, Autonomous University of Barcelona (UAB), 08193 Cerdanyola del Vallès, Spain

**Keywords:** Inclusion bodies, Aggregation, Recombinant protein, Leucine zippers, Jun, Fos, Purity

## Abstract

**Background:**

Inclusion bodies (IBs) are biologically active protein aggregates forming natural nanoparticles with a high stability and a slow-release behavior. Because of their nature, IBs have been explored to be used as biocatalysts, in tissue engineering, and also for human and animal therapies. To improve the production and biological efficiency of this nanomaterial, a wide range of aggregation tags have been evaluated. However, so far, the presence in the IBs of bacterial impurities such as lipids and other proteins coexisting with the recombinant product has been poorly studied. These impurities could strongly limit the potential of IB applications, being necessary to control the composition of these bacterial nanoparticles. Thus, we have explored the use of leucine zippers as alternative tags to promote not only aggregation but also the generation of a new type of IB-like protein nanoparticles with improved physicochemical properties.

**Results:**

Three different protein constructs, named GFP, J-GFP-F and J/F-GFP were engineered. J-GFP-F corresponded to a GFP flanked by two leucine zippers (Jun and Fos); J/F-GFP was formed coexpressing a GFP fused to Jun leucine zipper (J-GFP) and a GFP fused to a Fos leucine zipper (F-GFP); and, finally, GFP was used as a control without any tag. All of them were expressed in *Escherichia coli* and formed IBs, where the aggregation tendency was especially high for J/F-GFP. Moreover, those IBs formed by J-GFP-F and J/F-GFP constructs were smaller, rougher, and more amorphous than GFP ones, increasing surface/mass ratio and, therefore, surface for protein release. Although the lipid and carbohydrate content were not reduced with the addition of leucine zippers, interesting differences were observed in the protein specific activity and conformation with the addition of Jun and Fos. Moreover, J-GFP-F and J/F-GFP nanoparticles were purer than GFP IBs in terms of protein content.

**Conclusions:**

This study proved that the use of leucine zippers strategy allows the formation of IBs with an increased aggregation ratio and protein purity, as we observed with the J/F-GFP approach, and the formation of IBs with a higher specific activity, in the case of J-GFP-F IBs. Thus, overall, the use of leucine zippers seems to be a good system for the production of IBs with more promising characteristics useful for pharma or biotech applications.

## Background

Inclusion bodies (IBs) are protein nanoparticles ranging from 50 to 800 nm formed during the production of recombinant proteins and often localized in the cytoplasmic space of bacterial cells [1], although their formation in the periplasm has also been described [2]. In the past, IBs have been regarded as an undesired byproduct of recombinant protein production processes [3]. However, over the last 15 years it has been broadly proven that proteins forming IBs are biologically active [4–7]. The presence of native proteins forming such aggregates has prompted to assess their potential as biomaterials for a wide range of applications, including biocatalysis, tissue engineering, and antimicrobial and cancer therapies [7–10]. They offer important advantages over their soluble counterparts such as high stability [11], slow-release behavior [12, 13], and production through cost-effective processes [14]. Aiming to improve the production and biological efficiency of this nanomaterial, the increase of the aggregation tendency of proteins of interest is a key aspect and has been evaluated through the use of different aggregation tags, being VP1 [6], GFIL8 [15] and ELK16 [16] three representative examples. Besides, the scale-up of IB production and purification protocols have also been improved during the last decade [14, 17]. It has been described that the recombinant protein forming this biomaterial coexists with other proteins such as chaperones, and also with lipids [18, 19]. However, the presence of these impurities has been poorly studied, and consequently there is room to optimize IB composition. This is an important challenge to be solved in terms of IB applicability, since for specific applications it is important to control the exact composition of these bacterial nanoparticles. Thus, exploring ways to control the IB formation process emerges as a central strategy to improve IB purity and better control their physicochemical properties. In the present study, we have used an alternative approach for the generation of a new type of IB-like protein nanoparticles using leucine zippers (LZ) as aggregation-seeding domains with the aim to drive protein aggregation and improve IB properties for industrial applications. In marked contrast to aggregation tags used till present to increase protein aggregation, LZ are protein–protein interaction domains consisting of amphipathic α helices that dimerize in parallel, either as homodimers or heterodimers, to form a coiled-coil [20–22]. LZ dimerization motifs have already been explored as protein–protein interaction drivers both in recombinant mammalian [23] and bacterial [24, 25] cells. Thus, LZ specific properties make these peptides promising players to improve, control, and stabilize IB quality, thus boosting their potential for pharma and biotech industrial applications. In this study, we have explored the aggregation profile of a GFP reporter protein fused to Jun and Fos LZ [23] at different positions. The purity and formation of GFP IBs have been studied to determine the possible role of LZ in obtaining purer and better controlled IBs.

## Results

### Construct design and modelling

**Fig. 1:**
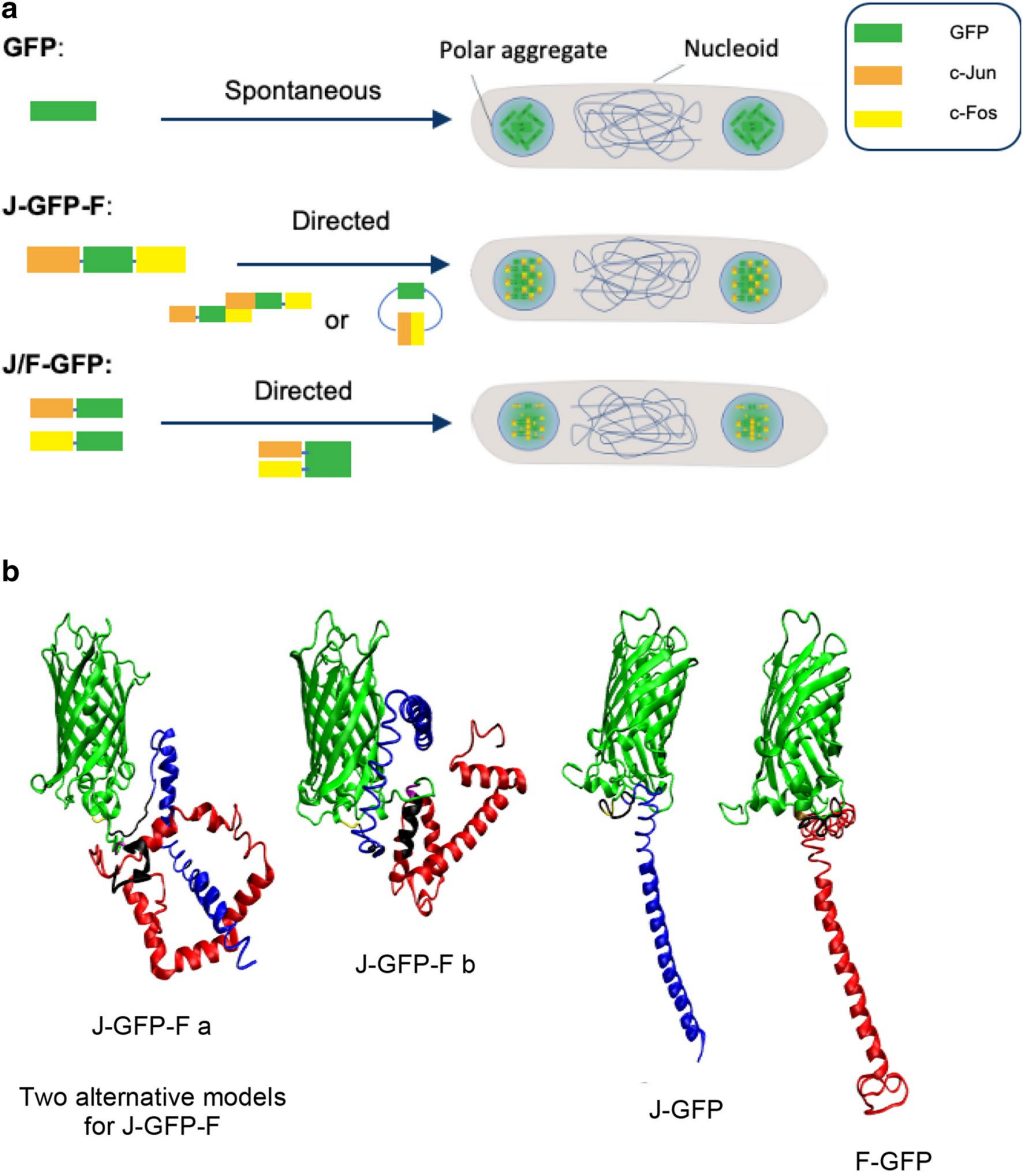
Recombinant construct. **a** Diagram of the protein-based constructs and their possible aggregation process as IBs depending on the presence or not of LZ. **b** Representative three-dimensional models of the J-GFP-F (two models: **a** and **b**) and J/F-GFP constructs (one model each), as generated by iterative threading. Construct domains are color coded as follow: GFP (green), Jun (blue), Fos (red)

In this study three different protein constructs, named GFP, J-GFP-F and J/F-GFP, have been engineered to evaluate the effect of Jun and Fos LZ on the protein aggregation process (Fig. 1a). J-GFP-F is a single fusion protein consisting on a GFP flanked by Jun and Fos at N- and C-terminus, respectively, whereas J/F-GFP is constituted by two proteins (a GFP with Jun LZ at N-terminus (J-GFP) and a GFP with Fos at N-terminus (F-GFP)), simultaneously coexpressed (Fig. 1). GFP protein has been used as a control without any LZ tag. The three-dimensional structural arrangements of the constructs were built by iterative threading, taking advantage of the I-TASSER webserver [26]. The generated models were visually inspected and possible three-dimensional arrangements of the J/F units with respect to the GFP were selected as representative conformational arrangements (Fig. 1b). In all constructs, the J/F subunits tended to assume a helical structure, as expected, but it was only possible to generate highly ordered starting domains for J/F-GFP. All models underwent 250 ns of atomistic molecular dynamics simulations in full water solvent (Additional file 1: Figure S1) showing the J/F elongated domains to be structurally unstable leading to partially disordered arrangements similar to those initially modelled for J-GFP-F. Among the generated models for J-GFP-F, no highly ordered structure was present, and the pool differed by the local arrangement of the J/F subdomains (as exemplified by two structures in Fig. 1b).

### Protein production and aggregation

**Fig. 2:**
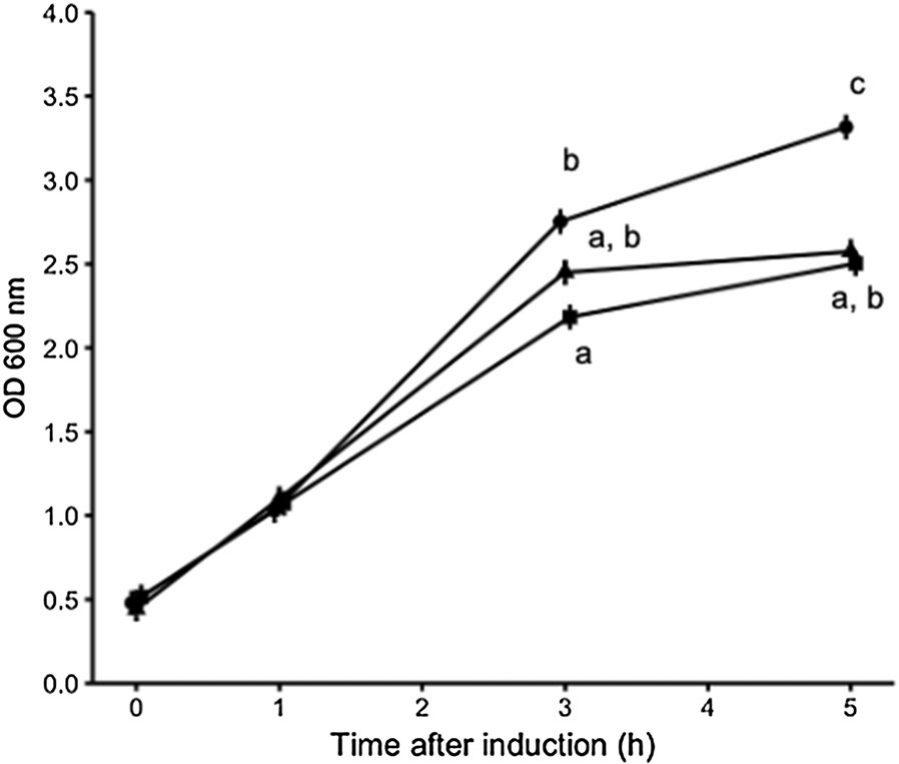
Optical density values of recombinant bacteria cultures in LB medium after protein expression induction. Circles represent *E. coli* pET22b/GFP (GFP), triangles *E. coli* pET22b/Jun-GFP-Fos (J-GFP-F), and squares represent *E. coli* pETDuet-1-Jun-GFP/Fos-GFP (J/F-GFP). Different letters depict significant differences between the growth curves (*p *≤ 0.0001)

Cell growth was determined under the overexpression of the three different constructs used in this study (Fig. 2). Interestingly, a reduction in the bacterial growth was observed when J-GFP-F and J/F-GFP were produced, especially at 5 h (Fig. 2). Moreover, those proteins that impaired the growth (J-GFP-F and J/F-GFP) were produced at lower levels than their GFP counterpart (Table 1). Analyzing the protein yields at different production time points, it can be observed that there is a time-dependent production of both GFP and J/F-GFP, while for J-GFP-F production values kept similar at different times post-induction (Table 1).

**Table 1:** Total protein yield (μg) of each recombinant construct at different times post-induction (1, 3, and 5 h)

	GFP			J-GFP-F			J/F-GFP			*p* value		
Time (h)	1	3	5	1	3	5	1	3	5	Treat	Time	Treat × time
Protein yield (μg)	52.39 ± 0.79	99.43 ± 1.03	223.82 ± 1.48	1.44 ± 0.12	1.23 ± 0.08	0.93 ± 0.07	5.12 ± 0.23	9.98±0.2	20.66 ± 1.96	< 0.0001	0.029	0.014

**Fig. 3:**
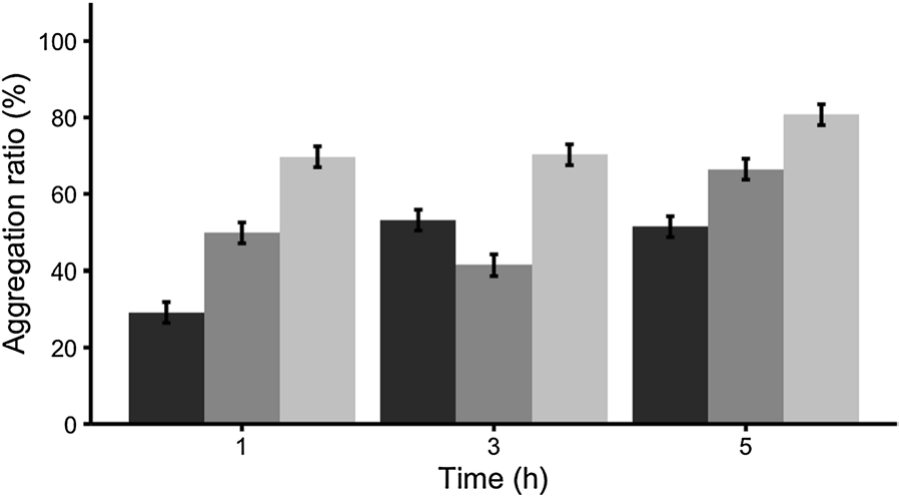
Protein aggregation ratio (%) for each construct over time. Black, dark grey, and light grey bars represent GFP, J-GFP-F, and J/F-GFP, respectively. Significant differences for construct (*p* ≤ 0.05) and for time (*p* ≤ 0.1; Additional file 1: Table S1)

Despite of the reduction in protein levels and in cell viability for those proteins carrying Jun and Fos (Fig. 2 and Table 1), all constructs aggregated (Fig. 3). Specifically, the analysis of the aggregation ratio showed differences among the constructs used, being the aggregation ratio higher for J/F-GFP than for GFP and J-GFP-F (Fig. 3 and Additional file 1: Table S1).

### Characteristics of purified IBs

**Fig. 4:**
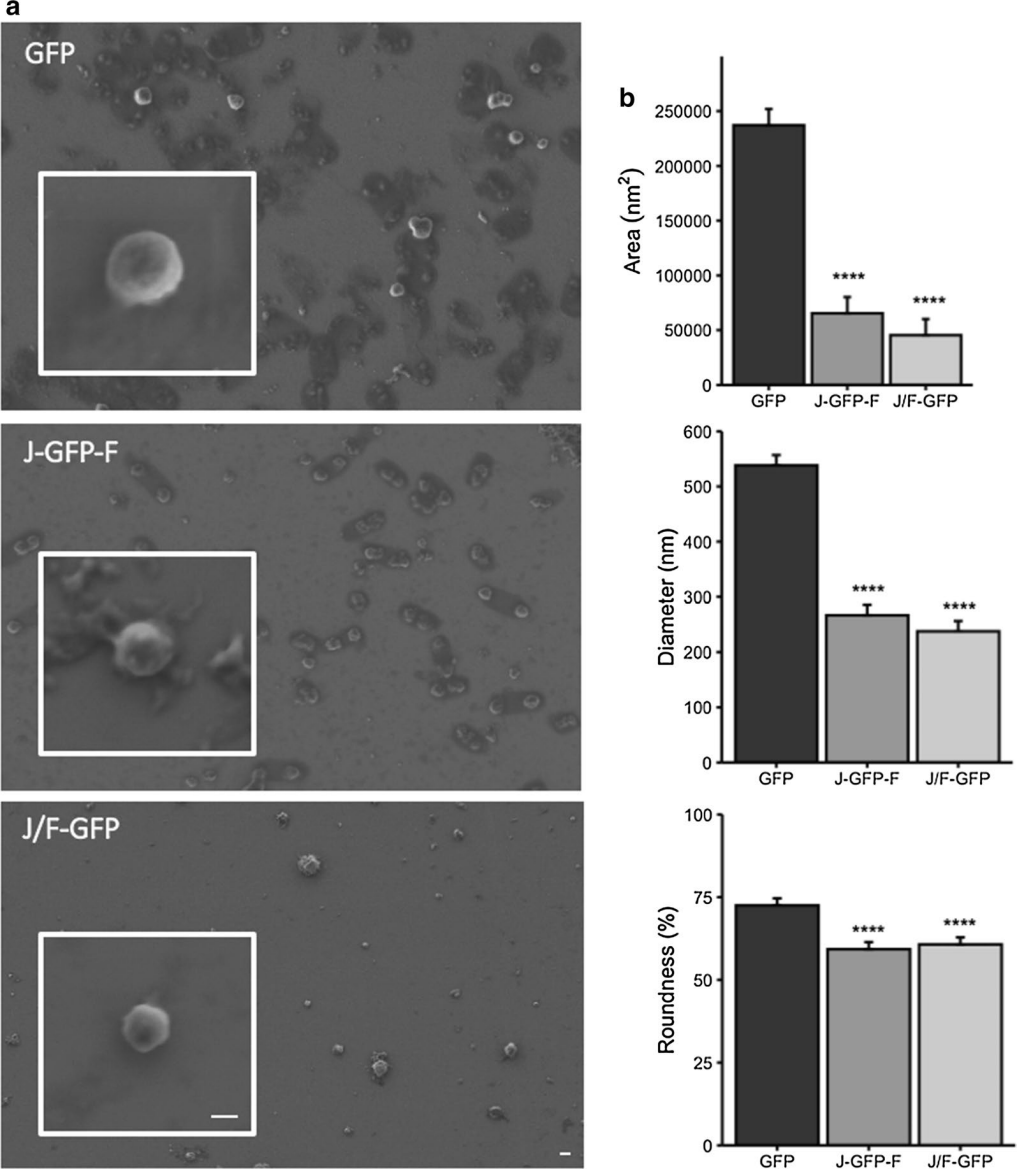
Representative FESEM images of the isolated IBs for each construct: GFP IBs, J-GFP-F IBs and J/F-GFP IBs. Bars size: 200 nm. IB mean area (nm^2^), mean diameter (nm) and roundness (%) was calculated for each construct IB (****p ≤ 0.0001)

Aiming to explore the specific nanoarchitectural characteristics of the protein nanoparticles formed using the three different tag combinations, IBs of each construct were purified. In all samples, qualitative and quantitative approaches with high resolution electron microscopy imaging of the ultrastructural morphometric of IBs showed a high number of well-formed nanoparticles with round shape and nanoscale size in the three constructs (Fig. 4). However, slight and important shape and size differences between GFP IBs and those nanoparticles formed with Jun or Fos LZ were detected. First of all, control GFP IBs showed a very homogeneous round shape and smooth surface, suggesting a highly compact structure (Fig. 4). In contrast, Jun-Fos based IBs showed a high variability in shape, significantly more amorphous than GFP ones and with a high percentage of IBs showing rough and porous surface. About the size, GFP nanoparticles showed the highest size with a diameter of 400–500 nm and a large mean area (Fig. 4), whereas Jun-GFP-Fos and Jun-GFP/Fos-GFP IBs were significantly smaller with a lower area and a diameter around 250 nm in both cases (Fig. 4).

**Fig. 5:**
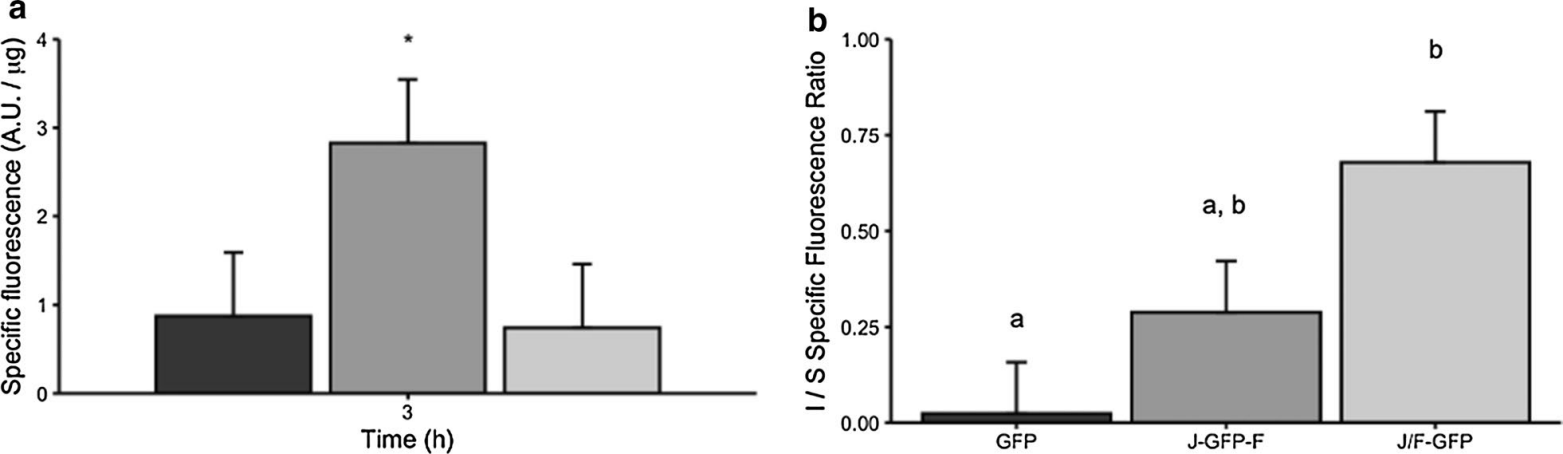
Specific fluorescence of the protein constructs. IB specific fluorescence for the three constructs (**a**). Specific fluorescence ratio of the insoluble fraction compared to the soluble fraction specific fluorescence (**b**). Black, dark grey, and light grey bars represent GFP, J-GFP-F, and J/F-GFP, respectively. * shows statistically significant differences compared to the GFP construct (*p* ≤ 0.05), and different letters also show statistically significant differences (*p* ≤ 0.05)

The analysis of the protein quality in terms of protein activity of GFP, J-GFP-F, and J/F-GFP IBs indicated that the addition of Jun and Fos improved the functional protein content when flanking the GFP, whereas no differences were observed for J/F-GFP compared to GFP (Fig. 5a). This indicates that the GFP forming J-GFP-F nanoparticles has better quality and, in consequence, higher fluorescence per μg of protein than the two other constructs used. Interestingly, the comparison of the specific fluorescence of GFP forming the IBs with the soluble version of this protein indicated that although the activity of the soluble form of all the proteins was higher than when forming IBs, the difference between soluble and IB-forming proteins was especially greater in the case of GFP (Fig. 5b).

**Fig. 6:**
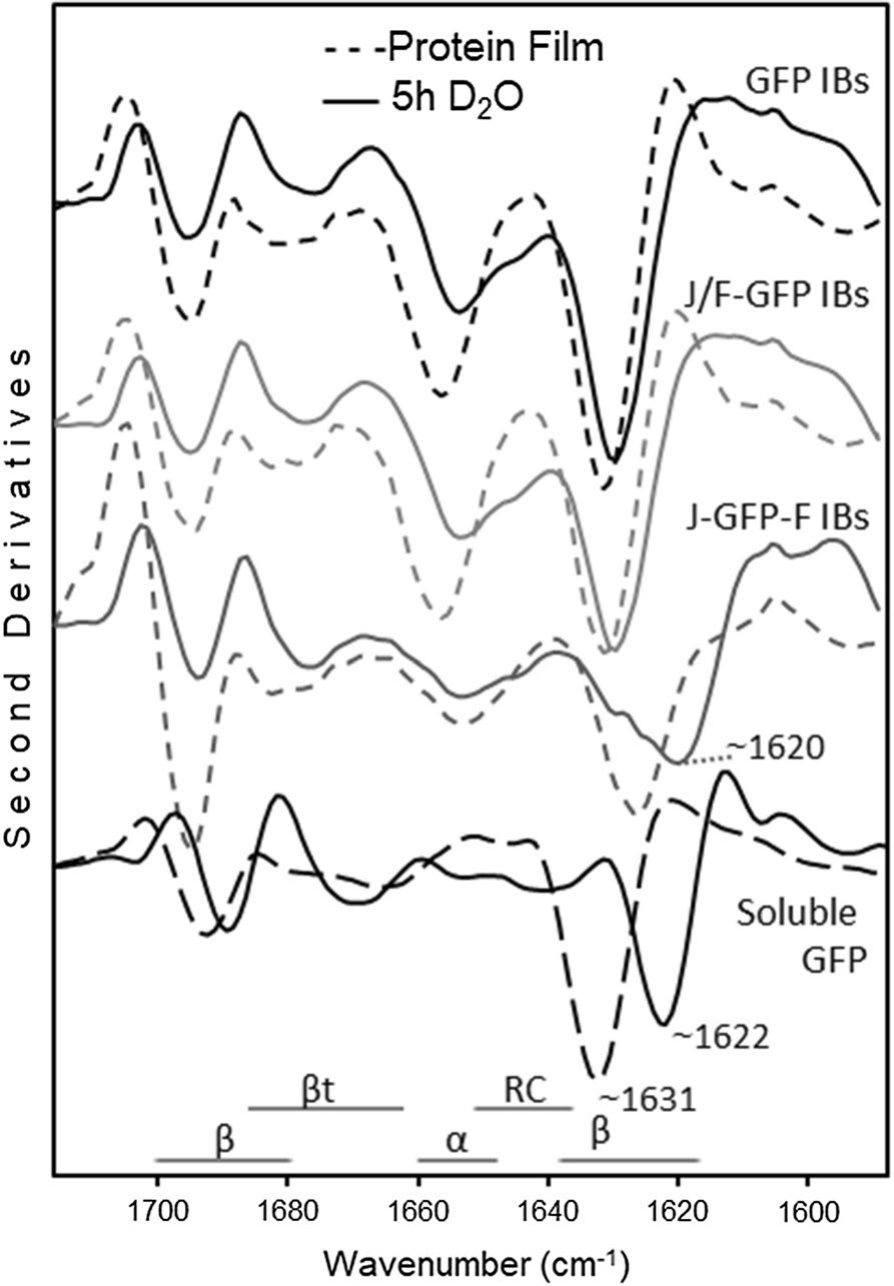
Second derivatives of the FTIR absorption spectra of GFP, J-GFP-F and J/F-GFP IBs and of soluble GFP. Samples were measured in form of protein films, obtained by solvent evaporation, and after re-hydration by D_2_O in order to allow H/D exchange. Selected peaks and the typical spectral region of the different protein secondary structures after H/D exchange are indicated. α, α-helices; β, β-sheets; βt, β-turns; RC, random coils

The conformational properties of the proteins embedded in the IBs were investigated by Fourier transform infrared (FTIR) spectroscopy [27]. The spectra were collected before and after hydrogen/deuterium (H/D) exchange to allow a better assignment of the Amide I band components to the protein secondary structures [28, 29]. GFP and J/F-GFP IBs displayed comparable IR response, while J-GFP-F IBs showed distinct peak positions and relative intensities of the spectral components assigned to β-sheets (Fig. 6 and Additional file 1: Figure S3). In particular, the absorption spectra (both before and after H/D exchange) of J-GFP-F IBs were more similar to those of the soluble GFP (Additional file 1: Figure S3) and the main β-sheet peak showed a higher downshift after H/D exchange compared to GFP and J/F-GFP IBs (Fig. 6). In the deuterated J-GFP-F, this component occurred at around 1620 cm^−1^, a peak position near to that observed for the main β-sheet band of soluble GFP, which indeed was observed around 1622 cm^−1^ (Fig. 6) [30, 31]. Overall, these data suggested that the Jun and Fos motifs modulated the conformational features of the formed IBs.

### Purity of IBs

**Fig. 7:**
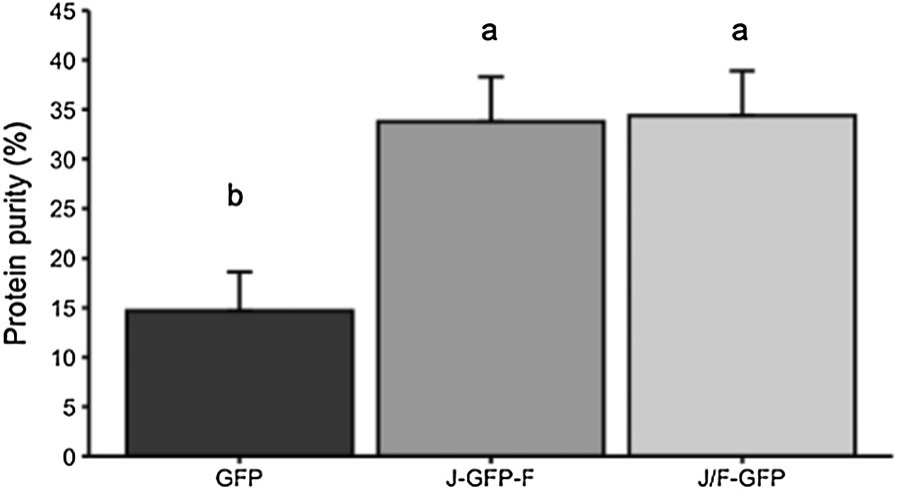
Protein purity of IBs. For each construct, protein purity was determined by densiometric image-analysis, comparing the amount of the specific recombinant construct to the whole protein amount of the IB. Different letters depict statistically significant differences (*p *≤0.05)

**Fig. 8:**
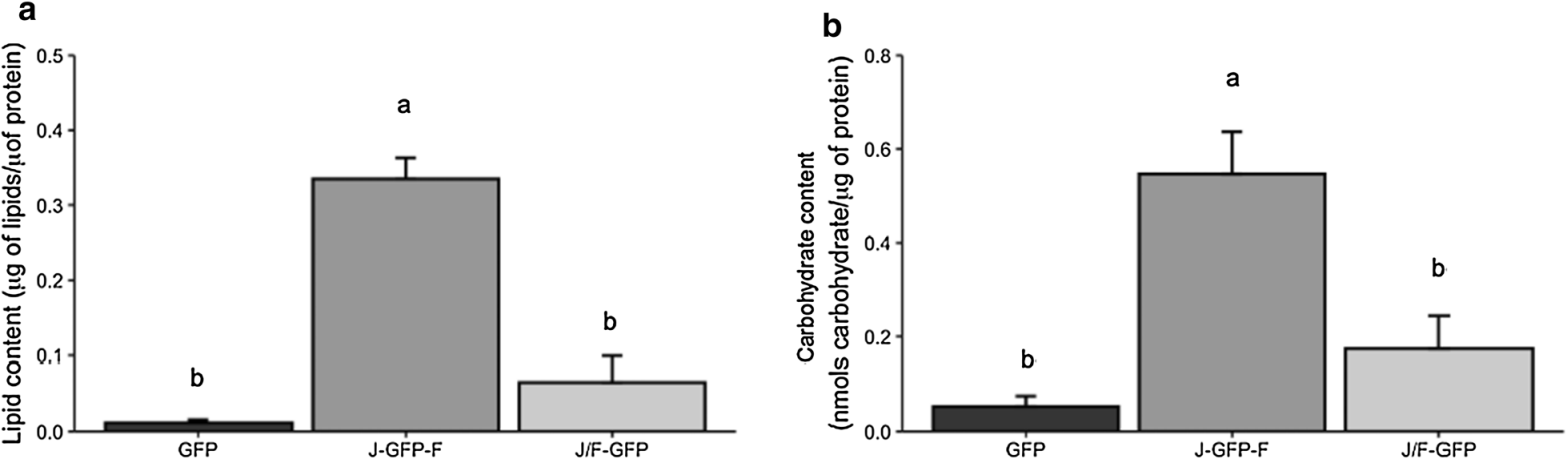
Lipid (**a**) and carbohydrate (**b**) content of for each IB construct. Different letters indicate statistically significant differences (*p* ≤0.01 (*a*) and *p* ≤0.05, (*b*))

Interestingly, J-GFP-F and J/F-GFP nanoparticles have a degree of protein purity more than twice than that observed for parental GFP nanoparticles (Fig. 7). Besides, the analysis of lipid and carbohydrate content in IBs indicated that the presence of Jun and Fos in J-GFP-F increased the presence of these co-contaminants while the strategy for the production of IBs using J/F-GFP showed no significant differences with GFP in lipid and carbohydrate content (Fig. 8).

## Discussion

IBs are protein aggregates ranging at nanoscale that have been widely studied from different perspectives. Since most of the recombinant proteins form IBs when overexpressed in bacteria [3], and some of them are only produced in this insoluble format, these nanoparticles have been broadly used as a source to obtain the soluble form of a wide range of protein of interest [32]. On the other hand, by being protein nanoparticles rich in functional recombinant protein, IBs have also been explored as a new class of biomaterial with promising applications in biocatalysis, tissue engineering, and human and animal therapies [7–9, 11]. However, although different aggregation tags have been used to promote their formation, the impact of these aggregation domains in the quality of protein aggregates has not been addressed so far. For that, in this work, we have explored if the use of Jun and Fos LZ dimerization motifs could drive the formation of IBs in a more controlled way in terms of quality. The results obtained proved that the constructs containing Jun and Fos (Fig. 1a), although they showed some toxicity to the producer cells (Fig. 2), they were produced as IBs with aggregation ratios higher than GFP (Fig. 3), proving that the aggregation propensity can be improved by using this strategy. The enhancement of the aggregation propensity can be attributed to the formation of Jun and Fos interactions among constructs, confirming that while the Jun and Fos domains seem to explore a number of partially disordered conformations when free in solution, this does not hinder the formation of stable aggregates. Interestingly, the aggregation ratio was observed to be higher for J/F-GFP than for J-GFP-F (Fig. 3). This can be rationalized in terms of competition between intramolecular and intermolecular Jun/Fos interactions. In J-GFP-F the Jun and Fos domains are entangled due to intramolecular interactions between them (Additional file 1: Figure S1). Their association within the same construct competes with the formation of dimers, or multimers, where Jun and Fos fragments belonging to different molecules interact and bond with each other. In both cases the two interacting fragments are the same, thus their energetic is expected to be close. At room temperature we might expect the competition and coexistence of monomers, dimers, and multimers in solution. However, it is also worth noting that in J-GFP-F, each Jun unit will be close to a Fos domain; this corresponds to a high local concentration of the partner, thus favoring the intramolecular entangled J-GFP-F. On the other hand, when the Jun/Fos domains are uncoupled, independently bound to different GFPs (as in J/F-GFP), there is obviously no competition between intermolecular and intramolecular interactions.

The study of the specific physicochemical characteristics of purified GFP, J-GFP-F and J/F-GFP IBs showed that, depending on the LZ strategy used, it’s possible to modulate IBs features such as specific activity, size, protein purity and presence of contaminants such as lipids and carbohydrates. The presence of LZ has a significant impact on both the size and shape of the aggregates, with IB diameters around 250 nm and more amorphous forms, while GFP IBs have a size of 400–500 nm and a higher average surface and higher roughness (Fig. 4). However, despite the differences in size, which can be correlated with the lower production yields achieved for those constructs containing LZ (Table 1), surface rugosity and roundness, ultrastructural morphometry of J-GFP-F and J-/F-GFP IBs (Fig. 4) agree with other conventional IBs produced in *E. coli* [33] and other cell factories such as *Lactococcus lactis* [34] or *Pichia pastoris* [35]. Interestingly, more amorphous shape and rough surface of J-GFP-F and J/F-GFP IBs could be indicative of significant differences in protein production and nanoparticle formation. In fact, the nanoarchitectural aspect of GFP IBs shows nanostructures with higher size, compactness and smooth surface than Jun and Fos IBs, which appear as more soft particles with a rough surface, more amorphous shape, and lower mean size. These nanoscale differences can play a great role in differential functionalities of proteins forming IBs in potential therapeutical applications. Then, low size, amorphous shape, and rough surfaces increase surface/mass ratio and, therefore, potential protein release at in vitro and in vivo conditions, becoming suitable and desired morphometric characteristics for more efficiently releasing nano or micro-platforms of drug delivery systems. Moreover, although smaller, GFP proteins forming J-GFP-F nanoparticles had a specific activity significantly higher than the other constructs that we tested (Fig. 5a), which is in accordance with FTIR spectra (Fig. 6 and Additional file 1: Figure S3). In particular, after H/D exchange, the main β-sheet peak of J-GFP-F IBs was observed to be very close to that observed for the soluble GFP, indicating the maintenance of native-like conformational features. Although J/F-GFP IBs showed no differences in the quality of the protein forming the IBs when compared to GFP (Fig. 5a), both J-GFP-F and J/F-GFP proteins forming IBs presented activities closer to the soluble form than GFP produced without tags (Fig. 5b). This indicates that the presence of Jun and Fos had a positive impact on the IB protein quality. This result agrees with previous studies that have described that the recombinant protein produced or the strain used can have an impact on the conformational quality of the proteins forming IBs [36–38].

On the other hand, the analysis of the elements forming such aggregates showed that J-GFP-F and J/F-GFP IBs had less protein impurities (Fig. 7), which suggests that Jun and Fos sequences drive a more controlled formation of the protein nanoparticles in terms of protein composition. By contrast, the presence of lipids and carbohydrates could not be decreased by the addition of LZ (Fig. 8). The amount of both lipids and carbohydrates in J/F-GFP IBs was comparable to the levels in GFP IBs, whereas in the case of J-GFP-F was even higher (Fig. 8). This could be correlated with the LZ interactions in each construct. As previously discussed, in J-GFP-F the Jun and Fos domains of different molecules compete with intramolecular interactions between the two domains, and this could drive the formation of less compact aggregates which could contain more lipids and carbohydrates.

Overall, these results demonstrate that aggregation-seeding domains based on LZ peptide–peptide interaction can drive the formation of a specific type of IBs, improving their quality in terms of protein content (Fig. 7), and in one of the approaches increasing specific activity (Figs. 5a and 6). However, those IBs with higher specific activity (J-GFP-F) are produced through a strategy that has a negative impact in the presence of contaminants such as lipids and carbohydrates (Fig. 8). On the contrary, the strategy based on the coexpression of Jun-GFP and Fos-GFP (J/F-GFP) to form IBs, even though it did not display any increase in the protein quality (Fig. 5a), had no negative impact in the content of lipids and carbohydrates (Fig. 8), therefore showing that it could be a promising approach for the production of IBs with higher recombinant protein content and less protein impurities.

## Conclusions

Altogether this study proved that the use of Jun and Fos LZ is a good strategy for the production of IBs with more appealing characteristics that might be useful for pharma or biotech applications. This is especially relevant for J/F-GFP approach, which allowed producing hybrid IBs with increased aggregation ratio and protein purity without negatively affecting their activity and lipid and carbohydrate content.

## Methods

### Construction of expression plasmids

The sequence encoding amino acid residues 2–238 of the enhanced GFP (EGFP) was fused downstream of sequence encoding Fos 118–210 (bFos) or Jun 257–318 (bJun) using the linker sequence encoding SGGGSGGS to construct Fos-GFP and Jun-GFP, respectively. For the Jun-GFP-Fos construct, the sequence encoding Jun 257–318 (bJun) was fused at the N-terminus, while the sequence encoding Fos 118–210 (bFos) was fused at the C-terminus, using in both cases the linker sequence encoding SGGGSGGS. The Jun-GFP-Fos construct was cloned into pET22b (Amp^R^) vector (pET22b-Jun-GFP-Fos), while Jun-GFP and Fos-GFP constructs were cloned in pETDuet-1 (Amp^R^) to co-express them (pETDuet-1-Jun-GFP/Fos-GFP). As a control, residues 2–238 of EGFP were cloned into pET22b. The DNA sequences corresponding to each gene sequence were codon optimized for its expression in *Escherichia coli* (GeneArt, Germany).

### Preparation of *E. coli* competent cells

*Escherichia coli* BL21 (DE3) cultures were grown overnight (ON) in LB medium at 37 °C with shaking at 250 rpm. A 1/100 inoculum was done in 50 ml of LB and the culture was grown until the optical density (OD_600nm_) reached a value between 0.2 and 0.4. After that, cultures were centrifuged (4000×*g*) at 4 °C for 15 min. Pellets were resuspended in 12.5 ml of cold and sterile 50 mM CaCl_2_ and incubated for 45 min in an ice bath. Cells were centrifuged again as described above and resuspended in 1.25 ml of cold and sterile 50 mM CaCl_2_ in glycerol (15% v/v) to prepare aliquots of 200 µl, which were stored at − 80 °C. To transform the cells, 40 ng of plasmid DNA were added to 200 µl of competent cells. The mixtures were incubated on ice for 30–60 min, warmed up to 42 °C for 45 s and placed on ice for 30 s. After incubation, 800 µl of LB media were added, and transformed cells were incubated at 37 °C for 1 h. Finally, the cells were plated on LB-agar plates containing the corresponding antibiotic.

### Protein production

*Escherichia coli* BL21 (DE3)/pET22b-GFP, *E. coli* BL21(DE3)/pET22b-Jun-GFP-Fos (J-GFP-F) and *E. coli* BL21(DE3)/pETDuet-1-Jun-GFP/Fos-GFP (J/F-GFP) ON cultures were inoculated in 50 ml of LB media with 100 µg/ml ampicillin in 200-ml flasks at an initial OD_600nm_ = 0.05. Each culture was grown at 37 °C and 250 rpm until the OD_600nm_ was 0.5 and 1 mM isopropyl-β-d-thiogalactoside (IPTG) was added to induce recombinant protein expression. At times 0, 1, 3, and 5 h after IPTG induction, 1 ml samples were collected for protein fractioning. These cultures were performed by triplicate.

### Protein fractionation

Samples of 1 ml were harvested by centrifugation at 6000×*g* at 4 °C for 15 min and the pellet was resuspended in 0.5 ml phosphate buffered saline (PBS) supplemented with protease inhibitor (Complete EDTA-free, Roche, Switzerland) to prevent protein proteolysis. Then, ice-jacketed samples were disrupted by sonication (2 cycles of 1.5 min at 10% amplitude under 0.5 s cycles) (Branson Ultrasonic SA, Switzerland). These samples were centrifuged at 15,000×*g* and 4 °C for 15 min to separate soluble and insoluble fractions protein fractions. Samples were stored at − 80 °C.

### Protein determination

Soluble and insoluble protein fractions were analyzed by denaturing SDS-PAGE (15% acrylamide). Denaturing buffer (Laemli 4×: Tris base 1.28 g, glycerol 8 ml, SDS 1.6 g, β-mercaptoethanol 4 ml, urea 9.6 g in 20 ml) was added to the insoluble and soluble fractions to a final concentration of 1× (see *protein fractioning*). Soluble and insoluble protein fractions were boiled for 10 and 45 min, respectively. At that time, samples were loaded onto the gel. SDS-PAGE protein bands were transferred onto PVDF membranes and identified using a commercial anti-GFP antibody (1:1000, sc-9996, Santa Cruz Biotechnology, USA), followed by an incubation with a secondary ALP-conjugated anti-mouse IgG (whole molecule) antibody (1:20,000, A4313, Sigma-Aldrich, USA). The amounts of recombinant protein were estimated by comparison with known amounts (usually ranging from 125 to 1000 ng) of T22-GFP protein [36]. Protein bands were visualized with a solution of NBT/BCIP (B6404, Sigma-Aldrich, USA), and images were obtained using a Color Image Scanner. ImageJ software was used to perform densitometric analyses of the bands.

### Determination of specific fluorescence

Fluorescence intensity of the three constructs in 1 ml samples was determined in a Varian Cary Eclipse fluorescence spectrometer (Agilent Technologies, Australia) at excitation and emission wavelengths of 480 and 510 nm, respectively. Since protein amounts in 1 ml samples for each construct, fraction and time are known (see *protein determination*), specific fluorescence was calculated by dividing the fluorescence intensity by the amount of protein for each construct, time and fraction.

### Protein purity assessment

After separation, the gels were carefully transferred to a plastic tray filled with 200 ml of distilled water and agitated at 50 rpm to remove SDS traces. The staining solution (Coomassie Brilliant BlueR-250 Staining Solution, Bio-Rad, USA) was added into the gels (and incubated for 1 h at room temperature (RT) and revealed with destaining solution (50% H_2_O, 40% methanol, 10% acetic acid (v/v)) until bands were clearly visible. The gel images were acquired by a Color Image Scanner and analyzed with the ImageJ software.

### Protein aggregation ratio

The amount of recombinant protein of the soluble and insoluble fraction for each construct was determined as explained above. After that, the aggregation ratio was calculated by dividing the quantity of the insoluble fraction for each time, replicate and construct by the quantity of protein in the respective soluble fraction.

### Purification of protein nanoparticles

Bacterial cultures were processed 3 h post-induction through a combination of mechanical and enzymatic disruption methods. Protease inhibitors (Complete EDTA-free, Roche, Switzerland), phenylmethanesulphonylfluoride (PMSF) and lysozyme were added to the culture at a final concentration of 0.4 mM (Sigma-Aldrich, USA) and 1 µg/ml (Sigma-Aldrich, USA), respectively. After 2 h of incubation at 37 °C and 250 rpm the culture was centrifuged at 6000×*g* and resuspended in 30 ml of PBS supplemented with protease inhibitors (Complete EDTA-free, Roche, Switzerland). Then, the mixture was ice-jacketed and sonicated for 4 cycles of 1.5 min at 10% amplitude under 0.5 s cycles (Branson Ultrasonic SA, Switzerland). After sonication, the mixture was frozen ON at − 80 °C. The mixture was thawed and Triton X-100 was added (0.4% (v/v)) and incubated for 1 h at RT. After this treatment, the mixture was frozen at − 80 °C for 2 h and then thawed for several cycles until no viable bacterial growth was detected. After that, 125 µl of Nonidet P40 (NP-40) was added and incubated for 1 h at 4 °C. Then, DNA was removed with DNAse at a final concentration of 0.6 µg/ml and 0.6 mM MgSO_4_ for 1 h at 37 °C. Samples were centrifuged at 15,000×*g* for 15 min at 4 °C. Pellets containing IBs were washed with 25 ml lysis buffer (50 mM Tris–HCl pH 8, 100 mM NaCl, 1 mM EDTA and Triton X-100 0.5% (v/v)). Finally, pellets were centrifuged at 4 °C for 15 min and 15,000×*g* and stored at − 80 °C until analysis. The IBs were quantified by western blot using a monoclonal anti-GFP antibody (1:1000, sc-9996; Santa Cruz Biotechnology, USA). All incubations were done under agitation.

### Electron microscopy

Field Emission Scanning Electron Microscope (FESEM) was used to visualize the ultrastructural morphology (size and shape) of protein nanoparticles in a nearly native state. For that, protein samples were directly deposited over silicon wafers (Ted Pella, USA), air dried, and observed with a high resolution standard secondary electron detector through a FESEM Merlin (Zeiss, Germany) operating at 2 kV. As a quantitative morphometric measurement, the mean area of IBs for each construct was analyzed as estimator of size with the Image J software. The number of particles was 30, 9, 11, for GFP, J-GFP-F and J/F-GFP, respectively. Using mean area values, the mean diameter of each particles was calculated, and the diameter was also used as an estimator of size. The roundness of IB particles for each construct was also evaluated (n = 50/construct), as an assessment of particle shape.

### Total carbohydrate and lipid analysis

The total lipid amount in IBs was determined following a sulfo-phospho-vanillin colorimetric assay. Briefly, 500–1000 µl of each sample were centrifuged at 15,000×*g* and 4 °C for 15 min. The supernatants were removed, and each pellet was dried by means of a vacuum lyophilizer (SpeedVac™, ThermoFisher) and dissolved in 200 µl of chloroform in a capped glass tube. The chloroform was evaporated at 63 °C in a fume hood. To each tube, 2 ml of 18 M sulfuric acid were added. The samples were then incubated for 10 min in a boiling water bath. After that, each tube was cooled down in ice for 5 min. Five milliliters of phosphoric acid-vanillin reagent (100 ml of 85% (v/v) phosphoric acid and 0.12 g of vanillin, (Sigma-Aldrich, Germany)) were added to the tubes and incubated for 15 min at 37 °C. The tubes were cooled for 15 min in ice and absorbance was measured at 530 nm. For the standard curve, a range of 10-100 μg triolein (dissolved in chloroform) was used. To determine the total content of carbohydrates present in the IBs samples, a phenol–sulfuric assay was performed. A glucose standard was prepared ranging from 0 to 150 nanomols and 150-225 µl of IBs were centrifuged at 15,000×*g* and 4 °C for 15 min. The supernatant was removed, and each pellet was dried with a vacuum lyophilizer. A total of 150 μl of 18 M sulfuric acid was added, followed immediately by 30 μl of 5% phenol. Samples were incubated at 90 °C for 5 min and then cooled to RT, and absorbance was measured at 490 nm. All assays were performed in triplicate.

### Structure modelling

J-GFP-F and J/F-GFP were modelled by iterative threading, as implemented the I-TASSER server [26] without applying any additional restrains. Given the primary sequence of the three constructs the server allows generating possible three-dimensional models by multiple threading alignment. The quality of the models was assessed both by their C-score, which was negative in all cases, and visually. For J-GFP-F, both the first (C-score = − 1.86) and the second (C-score = − 2.36) modes were chosen as these models showed different arrangements of the Jun/Fos domains. For both J-GFP (C-score = − 1.68) and F-GFP (C-score = − 2.87) the first model was chosen.

### Molecular dynamics protocol

For all the systems, the free construct was minimized, placed in a cubic box with a water layer of 0.7 nm and Na^+^ Cl^-^ ions to neutralize the system, and a second minimization was performed. We used AMBER99SB-ILDN [37] force field and Simple Point Charge water. NVT and NPT equilibrations were run for 100 ps, followed by 250 ns NPT production run at 300 °K. The temperature was controlled with a modified Berendsen thermostat [38], the pressure with an isotropic Parrinello-Rahman at 1 bar. The iteration time step was set to 2 fs with the Verlet integrator and LINCS [39] constraint. Periodic boundary conditions were used. All simulations and their analysis were run as implemented in the GROMACS package [40].

### FTIR analysis

IB samples were resuspended in a few hundred microliters of 20 mM of sodium phosphate buffer pH 7.4 and 2–3 µl of the suspension at 1 μg/μl were deposited in several drops on a BaF_2_ window and dried at RT in order to obtain a protein film. In particular, different sample concentrations were analyzed in order to obtain thin (to avoid excessive absorption) and uniform films without cracks [28]. Soluble GFP at 2 mg/ml in 20 mM sodium phosphate buffer pH 7.4, was analyzed in the form of a protein film as for IB samples. The transmission FTIR absorption spectra were then acquired by the Varian 670-IR FTIR spectrometer coupled to the Varian 610-IR infrared microscope (both from Varian, Australia) equipped with a mercury cadmium telluride nitrogen-cooled detector. The following conditions were employed: 2 cm^−1^ spectral resolution, 25 kHz scan speed, 512 scan co-additions, and triangular apodization. Several areas for each sample were measured to verify the reproducibility of the spectral results. Only absorption spectra with the Amide I band intensity below 0.8 were considered reliable. For H/D exchange, the protein film on the BaF_2_ window was rehydrated by the deposition of 8 μl of D_2_O around the dried film. The chamber was then tightly closed by a second window using a flat O-ring and incubated for 5 h at RT to allow H/D exchange [28, 34]. The FTIR spectra of the D_2_O-rehydrated samples were collected as described above.

Protein spectra were obtained after subtraction of the proper reference spectra strictly collected under the same conditions. The second derivatives [41] were calculated after spectral smoothing by the Savitsky-Golay method. Data collection and analysis were performed using the Resolutions-Pro software (Varian, Australia).

### Statistical analysis

All quantitative data are presented as mean values ± standard error of the mean (x̅ ± SEM). Normality of the data was determined by a Shapiro–Wilk test. For IB roundness, the data were normalized with the following formula: sqrt(max(x + 1) - x), where x is IB roundness and max the maximal roundness value in the data. A one-way ANOVA was conducted for all quantitative results except for IB surface and specific fluorescence measures. For the latter, values were compared with an independent sample *t*-test. Finally, we did Post hoc comparisons using the Tukey HSD test for all data analyzed by the ANOVA method. The level of significance was set at *p* < 0.05. Measures were done in triplicate, except for IB mean area, diameter and roundness, as indicated above. All statistical analyses were performed using the RStudio Statistical Software (RStudio, Inc., USA).

## Supplementary information

**Additional file 1.** **Figure S1**. Overlap between starting model (lighter shades) and final configuration (darker shades) of the J-GFP-F (two models: a and b) and J/F-GFP constructs (one model each), after 250 ns of molecular dynamics simulation. The generated models were minimized, placed in a cubic water box, minimized again, equilibrated and, for each construct, 250 ns of molecular dynamics simulation were performed. Large rearragements of the Jun/Fos domains were observed. Construct domains are color coded as follow: GFP (green), Jun (blue), Fos (red). **Figure S2**. (a) Representative FESEM images of the isolated IBs for each construct: GFP IBs, J-GFP-F IBs and J/F-GFP IBs. Bars size represent 200 nm. (b) Frequency distribution of IBs ultrastructural morphometry quantification for each construct: size (area (nm^2^) and diameter (nm)) and shape (roundness (%)). **Figure S3**. A) FTIR absorption spectra of the protein films. B) FTIR absorption spectra collected after re-hydration of the protein films with D_2_O for 5 h. GFP and J/F-GFP IBs displayed similar absorption spectra both as film and after re-hydration, while J-GFP-F IBs showed distinct spectral features. As a control, the absorption spectra of the soluble GFP are also shown. **Supplementary Table 1**. Statistics for the protein aggregation ratio (%) for each construct over time. (a) Aggregation ratio (%) differences between the three constructs and (b) aggregation ratio (%) differences for each construct over time. Different letters mean statistically significant difference (Post-hoc Tukey HSD (THSD) comparisons).

## Data Availability

The datasets used and/or analysed during the current study are available from the corresponding author on reasonable request.

## Abbreviations

LZ: Leucine zipper
IB: Inclusion body
GFP: Green fluorescent protein
IPTG: Isopropyl-β-d-thiogalactoside
FESEM: Field Emission Scanning Electron Microscope
H/D: Hydrogen/deuterium

## References

[1] García-Fruitós E, Sabate R, de Groot NS, Villaverde A, Ventura S (2011). Biological role of bacterial inclusion bodies: a model for amyloid aggregation. FEBS J.

[2] Choi JH, Lee SY (2004). Secretory and extracellular production of recombinant proteins using *Escherichia coli*. Appl Microbiol Biotechnol.

[3] Villaverde A, Corchero JL, Seras-Fanzoso J, Garcia-Fruitós E (2015). Functional protein aggregates: just the tip of the iceberg. Nanomedicine..

[4] García-Fruitós E, Vázquez E, Díez-Gil C, Corchero JL, Seras-Franzoso J, Ratera I, et al. Bacterial inclusion bodies: making gold from waste. Trends Biotechnol. 2012;30(2):65–70. https://linkinghub.elsevier.com/retrieve/pii/S0167779911001685. 10.1016/j.tibtech.2011.09.003 22037492

[5] Ventura S, Villaverde A. Protein quality in bacterial inclusion bodies. Trends Biotechnol. 2006;24(4):179–85. https://linkinghub.elsevier.com/retrieve/pii/S0167779906000527. 10.1016/j.tibtech.2006.02.007 16503059

[6] García-Fruitós E, González-Montalbán N, Morell M, Vera A, Ferraz RM, Arís A (2005). Aggregation as bacterial inclusion bodies does not imply inactivation of enzymes and fluorescent proteins. Microb Cell Fact.

[7] Rinas U, Garcia-Fruitós E, Corchero JL, Vázquez E, Seras-Franzoso J, Villaverde A. Bacterial Inclusion Bodies: Discovering Their Better Half. Trends Biochem Sci. 2017;42(9):726–37. https://linkinghub.elsevier.com/retrieve/pii/S0968000417300269. 10.1016/j.tibs.2017.01.005 28254353

[8] de Marco A, Ferrer-Miralles N, Garcia-Fruitós E, Mitraki A, Peternel S, Rinas U, et al. Bacterial inclusion bodies are industrially exploitable amyloids. FEMS Microbiol Rev. 2019;43(1):53–72. https://academic.oup.com/femsre/article/43/1/53/5144214. 10.1093/femsre/fuy038 30357330

[9] Pesarrodona M, Jauset T, Díaz-Riascos ZV, Sánchez-Chardi A, Beaulieu M, Seras-Franzoso J (2019). Targeting antitumoral proteins to breast cancer by local administration of functional inclusion bodies. Adv Sci..

[10] Roca-Pinilla R, López-Cano A, Saubi C, Garcia-Fruitós E, Arís A (2020). A new generation of recombinant polypeptides combines multiple protein domains for effective antimicrobial activity. Microb Cell Fact.

[11] Gifre-Renom L, Seras-Franzoso J, Rafael D, Andrade F, Cano-Garrido O, Martinez-Trucharte F (2020). The biological potential hidden in inclusion bodies. Pharm.

[12] Unzueta U, Cespedes MV, Sala R, Alamo P, Sánchez-Chardi A, Pesarrodona M, et al. Release of targeted protein nanoparticles from functional bacterial amyloids: A death star-like approach. J Control Release. 2018;279:29–39. https://linkinghub.elsevier.com/retrieve/pii/S0168365918301780. 10.1016/j.jconrel.2018.04.004 29641987

[13] Unzueta U, Seras-Franzoso J, Céspedes MV, Saccardo P, Cortés F, Rueda F, Garcia-Fruitós E, Ferrer-Miralles N, Mangues R, Vázquez E, Villaverde A (2017). Engineering tumor cell targeting in nanoscale amyloidal materials. Nanotechnology..

[14] Seras-Franzoso J, Peternel S, Cano-Garrido O, Villaverde A, García-Fruitós E (2015). Bacterial inclusion body purification.

[15] Wang X, Zhou B, Hu W, Zhao Q, Lin Z. Formation of active inclusion bodies induced by hydrophobic self-assembling peptide GFIL8. Microb Cell Fact. 2015 \;14(1):88. http://www.microbialcellfactories.com/content/14/1/88. 10.1186/s12934-015-0270-0 PMC4467046 26077447

[16] Wu W, Xing L, Zhou B, Lin Z (2011). Active protein aggregates induced by terminally attached self-assembling peptide ELK16 in *Escherichia coli*. Microb Cell Fact.

[17] Slouka C, Kopp J, Spadiut O, Herwig C (2019). Perspectives of inclusion bodies for bio-based products: curse or blessing?. Appl Microbiol Biotechnol.

[18] Rinas U, Bailey J (1992). Protein compositional analysis of inclusion bodies produced in recombinant *Escherichia coli*. Appl Microbiol Biotechnol.

[19] Fahnert B, Lilie H, Neubauer P (2004). Inclusion bodies: formation and utilisation.

[20] Hakoshima T (2005). Leucine Zippers. Encyclopedia of Life Sciences.

[21] Porte D, Oertel-Buchheit P, Granger-Schnarr M, Schnarr M (1995). Fos Leucine Zipper variants with increased association capacity. J Biol Chem.

[22] Vinson C, Myakishev M, Acharya A, Mir AA, Moll JR, Bonovich M. Classification of human B-ZIP proteins based on dimerization properties. Mol Cell Biol. 2002;22(18):6321–35. https://mcb.asm.org/content/22/18/6321. 10.1128/MCB.22.18.6321-6335.2002 PMC135624 12192032

[23] Hu C-D, Kerppola TK. Simultaneous visualization of multiple protein interactions in living cells using multicolor fluorescence complementation analysis. Nat Biotechnol. 2003;21(5):539–45. http://www.nature.com/articles/nbt816. 10.1038/nbt816 PMC1820765 12692560

[24] Choi S, Lee SJ, Yeom S, Kim HJ, Rhee YH, Jung H (2014). Controlled localization of functionally active proteins to inclusion bodies using Leucine Zippers. PLoS ONE.

[25] Han GH, Seong W, Fu Y, Yoon PK, Kim SK, Yeom SJ (2017). Leucine zipper-mediated targeting of multi-enzyme cascade reactions to inclusion bodies in *Escherichia coli* for enhanced production of 1-butanol. Metab Eng.

[26] Roy A, Kucukural A, Zhang Y. I-TASSER: a unified platform for automated protein structure and function prediction. Nat Protoc. 2010;5(4):725–38. http://www.nature.com/articles/nprot.2010.5. 10.1038/nprot.2010.5 PMC2849174 20360767

[27] Ami D, Natalello A, Taylor G, Tonon G, Maria Doglia S. Structural analysis of protein inclusion bodies by Fourier transform infrared microspectroscopy. Biochim Biophys Acta. 2006;1764(4):793–9. https://linkinghub.elsevier.com/retrieve/pii/S1570963905004401. 10.1016/j.bbapap.2005.12.005 16434245

[28] Natalello A, Doglia SM (2015). Insoluble protein assemblies characterized by Fourier transform infrared spectroscopy. Methods in molecular biology.

[29] Barth A (2007). Infrared spectroscopy of proteins. Biochim Biophys Acta - Bioenerg..

[30] Scheyhing CH, Meersman F, Ehrmann MA, Heremans K, Vogel RF (2002). Temperature-pressure stability of green fluorescent protein: a Fourier transform infrared spectroscopy study. Biopolymers.

[31] Tedeschi G, Mangiagalli M, Chmielewska S, Lotti M, Natalello A, Brocca S (2017). Aggregation properties of a disordered protein are tunable by pH and depend on its net charge per residue. Biochim Biophys Acta - Gen Subj..

[32] Singhvi P, Saneja A, Srichandan S, Panda AK. Bacterial inclusion bodies: a treasure trove of bioactive proteins. Trends Biotechnol. 2020;38(5):474–86. https://linkinghub.elsevier.com/retrieve/pii/S0167779919303063. 10.1016/j.tibtech.2019.12.011 31954528

[33] Cano-Garrido O, Rodríguez-Carmona E, Díez-Gil C, Vázquez E, Elizondo E, Cubarsi R, et al. Supramolecular organization of protein-releasing functional amyloids solved in bacterial inclusion bodies. Acta Biomater. 2013;9(4):6134–42. https://linkinghub.elsevier.com/retrieve/pii/S1742706112005831. 10.1016/j.actbio.2012.11.033 23220450

[34] Cano-Garrido O, Sánchez-Chardi A, Parés S, Giró I, Tatkiewicz WI, Ferrer-Miralles N (2016). Functional protein-based nanomaterial produced in microorganisms recognized as safe: a new platform for biotechnology. Acta Biomater.

[35] Rueda F, Gasser B, Sánchez-Chardi A, Roldán M, Villegas S, Puxbaum V (2016). Functional inclusion bodies produced in the yeast *Pichia pastoris*. Microb Cell Fact.

[36] Unzueta U, Céspedes MV, Ferrer-Miralles N, Casanova I, Cedano J, Corchero JL (2012). Intracellular CXCR4 + cell targeting with T22-empowered protein-only nanoparticles. Int J Nanomed..

[37] Lindorff-Larsen K, Piana S, Palmo K, Maragakis P, Klepeis JL, Dror RO (2010). Improved side-chain torsion potentials for the Amber ff99SB protein force field. Proteins Struct Funct Bioinforma..

[38] Bussi G, Donadio D, Parrinello M (2007). Canonical sampling through velocity rescaling. J Chem Phys..

[39] Hess B, Bekker H, Berendsen HJC, Fraaije JGEM (1997). LINCS: a linear constraint solver for molecular simulations. J Comput Chem.

[40] Pronk S, Páll S, Schulz R, Larsson P, Bjelkmar P, Apostolov R (2013). GROMACS 45: a high-throughput and highly parallel open source molecular simulation toolkit. Bioinformatics.

[41] Susi H, Byler DM (1986). Resolution-enhanced Fourier transform infrared spectroscopy of enzymes. Methods in enzymology.

