# Supplementary material for: Exploring the use of leucine zippers for the generation of a new class of inclusion bodies for pharma and biotechnological applications
Source: Microb Cell Fact. 2020 Sep 4;19:175. doi: 10.1186/s12934-020-01425-x (PMC7650227; doi:10.1186/s12934-020-01425-x)
Supplement: Supplementary file 1 — Additional file 1. Figure S1. Overlap between starting model (lighter shades) and final configuration (darker shades) of the J-GFP-F (two models: a and b) and J/F-GFP constructs (one model each), after 250 ns of molecular dynamics simulation. The generated models were minimized, placed in a cubic water box, minimized again, equilibrated and, for each construct, 250 ns of molecular dynamics simulation were performed. Large rearragements of the Jun/Fos domains were observed. Construct domains are color coded as follow: GFP (green), Jun (blue), Fos (red). Figure S2. (a) Representative FESEM images of the isolated IBs for each construct: GFP IBs, J-GFP-F IBs and J/F-GFP IBs. Bars size represent 200 nm. (b) Frequency distribution of IBs ultrastructural morphometry quantification for each construct: size (area (nm2) and diameter (nm)) and shape (roundness (%)). Figure S3. A) FTIR absorption spectra of the protein films. B) FTIR absorption spectra collected after re-hydration of the protein films with D2O for 5 h. GFP and J/F-GFP IBs displayed similar absorption spectra both as film and after re-hydration, while J-GFP-F IBs showed distinct spectral features. As a control, the absorption spectra of the soluble GFP are also shown. Supplementary Table 1. Statistics for the protein aggregation ratio (%) for each construct over time. (a) Aggregation ratio (%) differences between the three constructs and (b) aggregation ratio (%) differences for each construct over time. Different letters mean statistically significant difference (Post-hoc Tukey HSD (THSD) comparisons). [file 12934_2020_1425_MOESM1_ESM.docx]

Supplementary information

**Exploring the use of leucine zippers for the generation of a new class of inclusion bodies for pharma and biotechnological applications**

Ramon Roca-Pinilla^a^, Sara Fortuna^b^, Antonino Natalello^c^, Alejandro Sánchez-Chardi^d,e^, Diletta Ami^c^, Anna Arís^a^*, Elena Garcia-Fruitós^a^*

^a^Department of Ruminant Production, Institute of Agriculture and Food Research (IRTA), 08140 Caldes de Montbui, Spain

^b^ Department of Chemical and Pharmaceutical Sciences, University of Trieste, Via L. Giorgieri 1, 34127 Trieste, Italy

^c^ Department of Biotechnology and Biosciences, University of Milano-Bicocca, 20126 Milan, Italy

^d^ Department of Evolutionary Biology, Ecology and Environmental Sciences, Faculty of Biology, University of Barcelona (UB). 08028 Barcelona, Spain

^e^ Microscopy Service, Autonomous University of Barcelona (UAB), 08193 Cerdanyola del Valles, Spain

*Corresponding authors. Tel: + 34 93 467 40 40; Fax: +34 93 467 40 42; E-mail: anna.aris@irta.cat, [elena.garcia@irta.cat](mailto:elena.garcia@irta.cat)

**Figures**


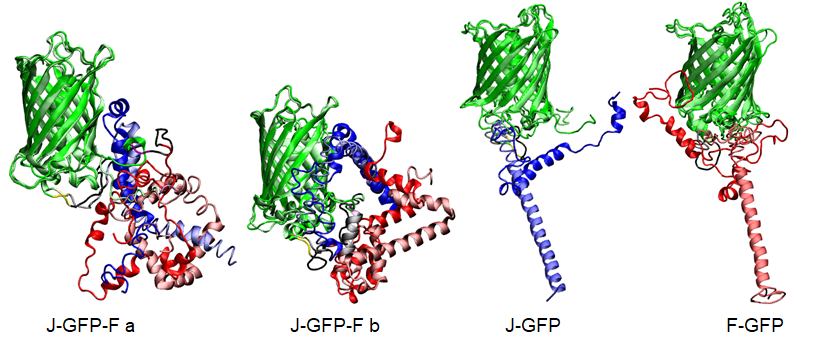


**Supplementary Figure S1.** Overlap between starting model (lighter shades) and final configuration (darker shades) of the J-GFP-F (two models: a and b) and J/F-GFP constructs (one model each), after 250 ns of molecular dynamics simulation. The generated models were minimized, placed in a cubic water box, minimized again, equilibrated and, for each construct, 250ns of molecular dynamics simulation were performed. Large rearragementsof the Jun/Fos domains were observed. Construct domains are color coded as follow: GFP (green), Jun (blue), Fos (red).


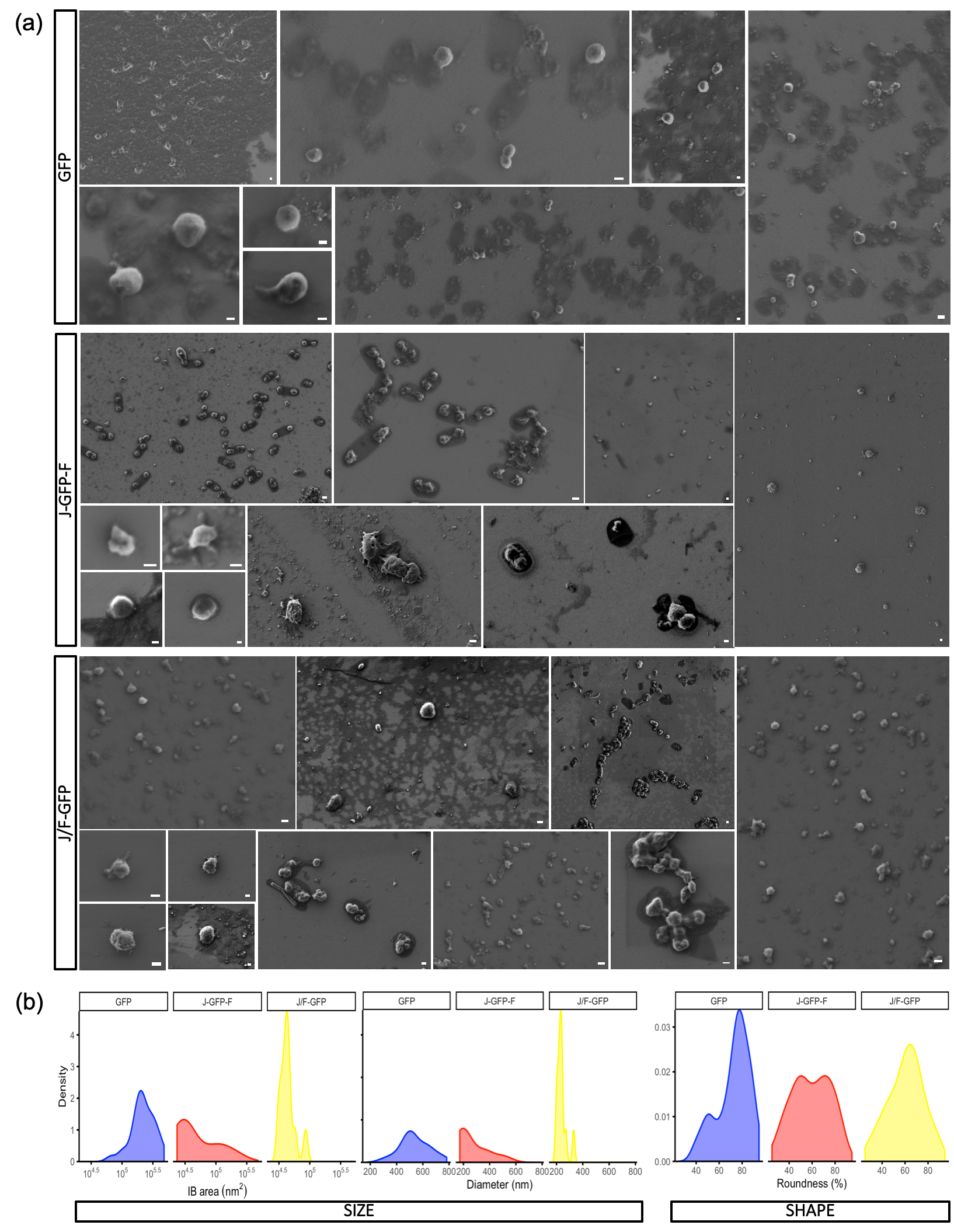


**Supplementary Figure S2. (**a) Representative FESEM images of the isolated IBs for each construct: GFP IBs, J-GFP-F IBs and J/F-GFP IBs. Bars size represent 200 nm. (b) Frequency distribution of IBs ultrastructural morphometry quantification for each construct: size (area (nm^2^) and diameter (nm)) and shape (roundness (%)).


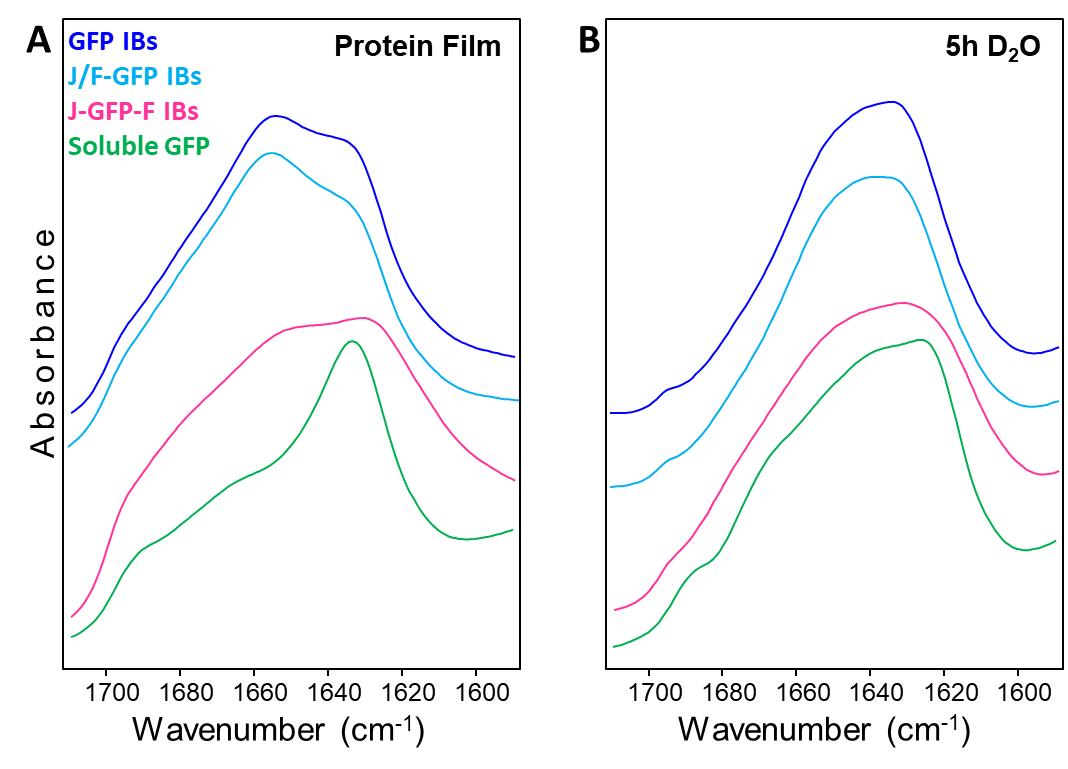


**Supplementary Figure S3.** A) FTIR absorption spectra of the protein films. B) FTIR absorption spectra collected after re-hydration of the protein films with D_2_O for 5 h. GFP and J/F-GFP IBs displayed similar absorption spectra both as film and after re-hydration, while J-GFP-F IBs showed distinct spectral features. As a control, the absorption spectra of the soluble GFP are also shown.

**Tables**

**Supplementary Table 1.** Statistics for the protein aggregation ratio (%) for each construct over time. (a) Aggregation ratio (%) differences between the three constructs and (b) aggregation ratio (%) differences for each construct over time. Different letters mean statistically significant difference (Post-hoc Tukey HSD (THSD) comparisons).

(a)

| **Protein** | **Aggregation ratio (%)** | **p-value** |
| --- | --- | --- |
| **GFP** | 44.57 ± 7.71 ^a^ | 0.0189 |
| **J-GFP-F** | 52.56 ± 7.36 ^a, b^ |  |
| **J/F-GFP** | 73.55 ± 3.59 ^b^ |  |

| **Protein** | **GFP** | | |  | **J-GFP-F** | | |  | **J/F-GFP** | | |  | p-value |
| --- | --- | --- | --- | --- | --- | --- | --- | --- | --- | --- | --- | --- | --- |
| **Time (h)** | **1** | **3** | **5** |  | **1** | **3** | **5** |  | **1** | **3** | **5** |  | Time |
| **Aggregation**  **ratio (%)** | 29.18±  17.15 ^a^ | 53.12±  18.85 ^a^ | 51.40±  3.80 ^a^ |  | 49.78±26.98 ^a^ | 41.43±30.11 ^a^ | 66.46±2.73 ^a^ |  | 69.71±15.15 ^a^ | 70.23±10.89 ^a^ | 70.23±9.14 ^a^ |  | 0.057 |

(b)
